# Dynamic succession of microbial compost communities and functions during *Pleurotus ostreatus* mushroom cropping on a short composting substrate

**DOI:** 10.3389/fmicb.2022.946777

**Published:** 2022-08-18

**Authors:** Qin Liu, Weili Kong, Xiao Cui, Sujuan Hu, Ziwen Shi, Jie Wu, Yuting Zhang, Liyou Qiu

**Affiliations:** ^1^Institute of Plant Nutrition, Agricultural Resources and Environmental Science, Henan Academy of Agricultural Sciences, Zhengzhou, China; ^2^College of Life Sciences, Henan Agricultural University, Zhengzhou, China

**Keywords:** composting substrate, dynamic change, metabolic function, microbial community, *Pleurotus ostreatus*

## Abstract

Cultivating oyster mushrooms (*Pleurotus ostreatus*), a typical primary decomposer of lignocellulose, on a short composting substrate is a novel procedure which possesses energy conserves, reduced the chance of infection by competitive species, shorter production duration and achieved high production efficiency. However, the microbiome and microbial metabolic functions in the composting substrate during the mushroom cropping is unknown. In the present study, the contents of hemicellulose, cellulose and lignin and the activities of protease, laccase and cellulase were evaluated in the corncob short composting substrate from before oyster mushroom spawning to first flush fructification; meanwhile the changes in the microbiome and microbial metabolic functions were surveyed by using metagenomic sequencing. Results showed that the hemicellulose, cellulose and lignin in the short composting substrate were decomposed of 42.76, 34.01, and 30.18%, respectively, during the oyster mushroom cropping process. In addition, the contents of hemicellulose, cellulose and lignin in the composting substrate were reduced rapidly and negatively correlated with the abundance of the Actinobacteria phylum. The activities of protease, laccase and cellulase fastly increased in the period of before oyster mushroom spawning to full colonization and were positively correlated to the abundance of Actinobacteria phylum. The total abundance of bacteria domain gradually decreased by only approximately 15%, while the abundance of Actinobacteria phylum increased by 68% and was positively correlated with that of oyster mushroom. The abundance of oyster mushroom increased by 50 times from spawning to first flush fructification. The dominant genera, all in the order of Actinomycetales, were *Cellulosimicrobium*, *Mycobacterium*, *Streptomyces* and *Saccharomonospora*. The total abundance of genes with functions annotated in the Clusters of Orthologous Groups of proteins (COG) for Bacteria and Archaea and Kyoto Encyclopedia of Genes and Genomes (KEGG) database for all three life domains was positively correlated.

The three metabolic pathways for carbohydrates, amino acids and energy were the primary enrichment pathways in KEGG pathway, accounting for more than 30% of all pathways, during the mushroom cropping in which the glycine metabolic pathway, carbon fixation pathways in prokaryotes and methane metabolism were all dominated by bacteria. The genes of cellulolytic enzymes, hemicellulolytic enzymes, laccase, chitinolytic enzymes, peptidoglycanlytic enzymes and ammonia assimilation enzymes with abundances from 0.28 to 0.24%, 0.05 to 0.02%, 0.02 to 0.01%, 0.14 to 0.08%, 0.39 to 0.16%, and 0.13 to 0.12% during the mushroom cropping identified in the Evolutionary Genealogy of Genes: Non-supervised Orthologous Groups (eggNOG) database for all three life domains were all aligned to COG database. These results indicated that bacteria, especially Actinomycetales, were the main metabolism participants in the short composting substrate during the oyster mushroom cropping. The relationship between oyster mushrooms and bacteria was cooperative, Actinomycetales were oyster mushroom growth promoting bacteria (OMGPB).

## Graphical Abstract

Metagenomic analysis revealed oyster mushroom growth-associated microbial communities. 
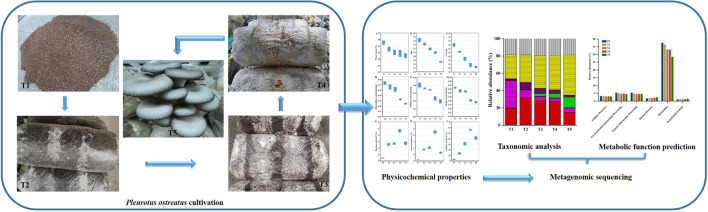

## Introduction

Oyster mushroom (*Pleurotus ostreatus*) is an edible mushroom that is frequently cultivated in China, ranking third in terms of annual production behind only *Lentinula edodes* and *Auricularia auricula* (Liu et al., 2020). The rich nutritional content, good flavor and hygienic function of oyster mushroom have made it popular among consumers (Kong et al., 2020). Oyster mushroom is a typical wood white-rot fungus and a primary decomposer of dead plant biomass (Bonatti et al., 2004; Rytioja et al., 2014) that can grow on logs and sawdust without the assistance of other bacteria and fungi and can be cultivated on pasteurized or sterilized lignocellulosic substrates. Additionally, oyster mushroom performs well as a straw rotting fungus and secondary decomposer, can utilize wheat and rice straw, corncob, rice husk and cotton seed hull substrates, and grows on composting substrates along with bacteria and other fungal species (Savoie et al., 2013; Zied et al., 2019). The versatility of oyster mushroom allows its cultivation using diverse raw materials and various treatments to prepare substrates.

Composting substrates have been employed worldwide to cultivate oyster mushrooms (Bánfi et al., 2015). In comparison with pasteurization or sterilization, composting conserves energy, protects the environment and is inexpensive; it also reduces the chance of subsequent infection by competitive species and achieves high production efficiency (Vajna et al., 2010; Kong et al., 2020). The microbial community structures associated with the composting substrate preparation processes of oyster mushrooms have been reported previously (Choi, 2004; Vajna et al., 2010, 2012; Vieira et al., 2019; Kong et al., 2020), but not in the context of the mushroom cropping. The microbial community structure and the relationship between mushroom and the microbe inhabited in compost have been extensively studied during the button mushroom (*Agaricus bisporus*) cropping. The button mushroom compost is composted over a long period of phase I and phase II composting. During phase II, the high concentrations of easily accessible nutrients and ammonia formed in phase I are used by thermophilic fungi and bacteria, among which *Mycothermus thermophilus* and *Pseudoxanthomonas* become the most dominant microbiota at the end of phase II (Natvig et al., 2015; Kertesz et al., 2016b). However, *Mycothermus thermophilus* and bacteria in the compost are vigorously decomposed by button mushroom in the course of the mushroom spawn running (Vos et al., 2017), whereas only *Pseudomonas* from Proteobacteria increase successively (de Boer et al., 2016; Kertesz et al., 2016a; Carrasco and Preston, 2020; Carrasco et al., 2020). Wherefore, the relationship between the button mushroom mycelia and microorganisms in the compost seems to indicate more conflict than cooperation (Kertesz and Thai, 2018).

The interactions between *Pseudomonas* in the compost and button mushroom during the mushroom cropping remain undefined. But *Pseudomonas* in the mushroom casing metabolizes 1-octene-3-ol (Noble et al., 2009) or 1-aminocylopropane-1-carboxylic acid (ACC), a precursor of ethylene (Chen et al., 2013; Zhang et al., 2016), and promotes the mushroom primordium formation, functions as mushroom growth promoting bacteria (MGPB) (Agnolucci et al., 2015). The other MGPB including some strains of *Bacillus*, Actinomycetales, *Staphylococcus* and *Bradyrhizobium* also promote the mycelial growth, accelerate primordial development and increase yield of *Agaricus* and *Pleurotus* mushrooms (Ahlawat and Vijay, 2010; Young et al., 2013; Zhu H. X. et al., 2013). Similarly, several soil bacterial strains of *Pseudomonas* (Duponnois and Garbaye, 1991; Deveau et al., 2007; Gamalero et al., 2008) and *Streptomyces* (Schrey et al., 2007) help mycorrhizas establishment or functionality, thence defined as mycorrhiza helper bacteria (MHB) by Garbaye (1994).

The use of a short composting substrate to cultivate oyster mushrooms has been strongly promoted in China. The short composting substrate is prepared during a short composting period, without phase II composting compared to the standard compost procedure of button mushroom, yielding compost with high levels of easily accessible nutrients and ammonia, resulting in high respiration activity, self-heating and ammonia effects during mushroom colonization (Kong et al., 2021). Furthermore, thermophilic fungi are not predominant in the composting substrate used for oyster mushrooms (Kong et al., 2020). Thus, the microbial community and population dynamics of such compost should be different from those of *Agaricus bisporus* over the duration of mushroom cropping. Previously, we reported shifts in bacterial community structure that occurred in the course of a short composting process (Kong et al., 2020). In the present study, we employed metagenomics approach to investigate the variations in the microbial population and functional genes during oyster mushroom cropping on the short composting substrate, and the role played by microorganisms resided in the composting substrate in substrate utilization. The primary goals were to reveal the relationship between the oyster mushroom and the microorganisms in the composting substrate, and explore the effective strategies to increase the yield and quality of oyster mushroom growing on the composting substrate.

## Materials and methods

### Oyster mushroom cultivation and sampling

The trial was conducted in the Modern Agricultural Science and Technology Experimental Demonstration Base of Henan Academy of Agricultural Sciences in Xinxiang, Henan Province, China. The composting substrate for cultivation of oyster mushroom was prepared as described in a previous report (Kong et al., 2020). After composting, the pile was spread out to let the heat dissipate. Polyethylene bags (51 cm × 26 cm × 0.0015 cm) and the layer spawning method were used to cultivate oyster mushroom. Each cultivation bag contained four layers of spawn and three layers of compost, with a total weight and spawning quantity of 4.0 kg and 15% (w/w), respectively. The cultivation cycle of oyster mushroom requires approximately 30 d and occurs in two successive phases (Sánchez, 2010). The first phase is the vegetative growth phase, in which the mycelia of oyster mushroom grow profusely in the composting substrate at 24 ± 1°C under conditions of 60–70% relative humidity in the dark. This phase is divided into several stages: composting substrate preparation, mycelium colonization, mid-incubation and end-incubation. The second phase is fructification and harvest, in which the umbrella-like body develops until the end of the cycle. During the second phase, the humidity, temperature, and light level were altered (85–95% relative humidity, 18°C, 100 lx scattered light) so that mushrooms would form and develop fruiting bodies (Chang and Wasser, 2017). Representative samples were collected at five time points during the entire cultivation period: T1 (day 0: composting completed), T2 (day 3: mycelial colonization), T3 (day 10: mid-incubation), T4 (day 20: end-incubation), and T5 (day 30: first oyster mushroom fructification). Composting substrate collected from at least nine cultivation bags on the sampling dates was homogenized together by hand to provide representative samples, which were separated into two parts. The first part of the sample was air-dried prior to the physicochemical analysis, and the second part of the sample was stored at –80°C.

### Physicochemical analysis

The samples were dried for 24 h at 105°C to a constant weight, which was used to determine the water content of the composting substrate. The pH was measured as described by Kong et al. (2020). Composting substrate (10 g, wet weight) was added to 50 mL distilled water, after which the resulting mixture was shaken for approximately 30 min to bring the samples to equilibrium. The pH of the mixture was then determined with a combination pH meter (PHS-3C, INESA Scientific Institute Co., Ltd., Shanghai, China). The potassium dichromate method was used to determine the organic matter content of each sample (Kong et al., 2020). The Kjeldahl method was used to measure the total nitrogen content of the composting substrate. The C/N ratio was determined as follows: C/N = (%C/%N). The contents of hemicellulose, cellulose and lignin were determined based on the National Agriculture Industry Standard (NY/T 3494-2019, China).

Ten grams of composting substrate was mixed with phosphate buffer (100 mL at 100 mmol L^–1^, pH 7.2). After oscillation on a rotary shaker for 1 h to extract the enzymes, the mixture was centrifuged at 9,000 *g* for 10 min at 4°C, after which the supernatant was collected and used to measure the activities of selected enzymes. Assays of protease activity were performed as reported previously by Wang and Ng (2001). One unit (U) of protease activity was determined to be the amount of enzyme that resulted in a change in absorbance of 0.001 per min at 280 nm. The laccase activity of each sample was measured as described in a previous report (Zhu M. J. et al., 2013). The reaction mixture contained 200 μL of enzyme solution and 400 μL of 1 mmol L^–1^ 2,2-azino-bis-(3-ethylbenzothiazoline-6-sulfonic acid) (ABTS) (Sigma-Aldrich, St. Louis, MO, United States) in sodium acetate buffer (10 mmol L^–1^, pH 4.6) that was incubated at 25°C for 10 min. One unit of laccase activity (U) was considered to be the amount of enzyme that increased the absorbance units by 1.0 per min at 405 nm. The cellulase activity was determined by incubating 100 μL of enzyme with 100 μL of carboxymethyl cellulose (Sinopharm Chemical Reagent Co., Ltd., Shanghai, China) solution (0.5%, w/v) in citrate acid buffer (100 mmol L^–1^, pH 5.0) for 30 min at 37°C. Next, 3,5-dinitrosalicylic acid (Sinopharm Chemical Reagent Co., Ltd., Shanghai, China) was applied to allow estimation of the total concentration of reducing sugars by measuring their absorbance at 540 nm and describing them as glucose equivalents (Qian et al., 2020). One unit of cellulase (U) was considered to be the amount of enzyme that caused the release of 1 μg reducing sugar per min.

### Deoxyribonucleic acid extraction, library preparation and sequencing

Total deoxyribonucleic acid (DNA) was extracted from each sample using commercial kits (soil sample: DP 336/stool sample DP328, Tiangen Biotech Co., Ltd., Beijing) following the manufacturer’s instructions. More than 1 μg DNA was extracted from each sample. An aliquot of extracted DNA was subjected to gel electrophoresis (0.8% agarose gel) to allow visualization of DNA quality and size; the bands were clear, suggesting no degradation or slight degradation. The extracted DNA (250 ng) was sonicated in a sonicator (JY92-IIN, Xinzhi, Ningbo, Zhejiang, China) to a fragment size ranging from 200 to 500 bp. Sequencing libraries were prepared using the VAHTS Universal DNA Library Prep Kit for Illumina^®^ (Catalog NO. ND607, Vazyme Biotech Co., Ltd., Nanjing, China) following the manufacturer’s instructions. PCR products corresponding to 200–500 bp were enriched, quantified and finally sequenced on a NovaSeq 6000 sequencer (Illumina Inc., San Diego, CA, United States).

### Metagenomic analysis

Residual adaptor sequences and leading and trailing bases with low quality scores were removed from the raw reads using Trimmomatic v0.36 (Bolger et al., 2014). FastQC v0.11.5 was used to evaluate the clean reads for quality and adaptor contamination (Andrews, 2010). Megahit v1.1.2 (Li et al., 2015) was used to stitch the filtered reads to obtain the most optimized contigs, in which open reading frames (ORFs) were predicted using Prokka v1.13.3 (Seemann, 2014). Amino acid sequences were translated from ORF sequences longer than 100 bp, and the resulting sequences were used to prepare a non-redundant gene set. All clean reads of each sample were mapped to the gene set using Bowtie2 v2.3.3.1 (Langmead and Salzberg, 2012), and the abundances of genes in each sample were calculated. The Shannon and Simpson indices were calculated, and genes with significantly different abundance were screened with the Wilcoxon rank-sum test. The gene set was annotated based on the KEGG, COG, Carbohydrate-Active Enzymes Database (CAZy) (Lombard et al., 2014), Antibiotic Resistance Genes Database (ARDB) (Liu and Pop, 2009) and Virulence Factors Database (VFDB) (Chen et al., 2005) using DIAMOND v0.9.10 (Buchfink et al., 2015). DIAMOND v0.9.10 was also used to execute the “blastp” command to map the amino acid sequences translated from the gene set against the National Center for Biotechnology Information (NCBI) “non-redundant database” to produce Diamond alignment archive (DAA) files, and the files were applied to obtain the gene function and homologous species information at E-value < 1e^–5^ by using MEGAN Community Edition (CE) (Huson et al., 2016). The relative abundances of species were calculated at taxonomic level of the kingdom, phylum, class, order, family and genus.

### Statistical analysis

Microsoft Excel 2016 (Microsoft Cooperation, Redmond, WA, United States) and Origin 2017 (OriginLab, Northampton, MA, United States) were used for all data generation, resulting in values that were expressed as the mean ± standard deviation. Statistical analyses of the physicochemical properties of the composting substrate and the relative abundance of members of its microbial community were performed using SPSS v. 20.0 (IBM, Inc., Armonk, New York, United States). One-way analysis of variance (ANOVA) was performed using Tukey’s test (*p* < 0.05 threshold for statistical significance). Pearson correlational analysis was used to determine the relationship between the composition of the microbial community and its physicochemical properties. Analysis and visualization were conducted using R (R Core Team, 2017), RStudio (RStudio Team, 2015), and the R packages corrplot (Wei and Simko, 2017), factoextra (Kassambara and Mundt, 2020), ggplot2 (Wickham, 2009), and Hmisc (Harrell, 2018). The R software vegan package (v. 3.1.2) was applied to determine the similarity among the microbial community composition in the composting substrate at the different growth stages of oyster mushroom by utilizing a non-metric multidimensional scaling (NMDS) analysis based on Bray-Curtis distance matrices and analysis of similarity (ANOSIM). The relative abundance of different functional hierarchies was considered to be equal to the sum of the relative abundance of genes annotated to that functional level. Version 2.1.3 of the vegan package of R software was also utilized to draw a heatmap of the relative abundance of the 50 most significant metabolic functions based on the level 3 KEGG ortholog functions. The linear discriminant analysis (LDA) effect size (LEfSe) (Segata et al., 2011) was used to identify differences in genomic features (genes, pathways, or taxa) among two or more groups. Based on the table of KEGG Orthology (KO) abundance, differences in the *p*-values of KOs between two groups were determined using the Wilcoxon rank sum test, and KOs with significant differences in expression between two groups were identified based on a threshold of *p* < 0.05. Calculations were conducted using STAMP (Parks et al., 2014).

## Results

### Physicochemical changes in composting substrate

**FIGURE 1:**
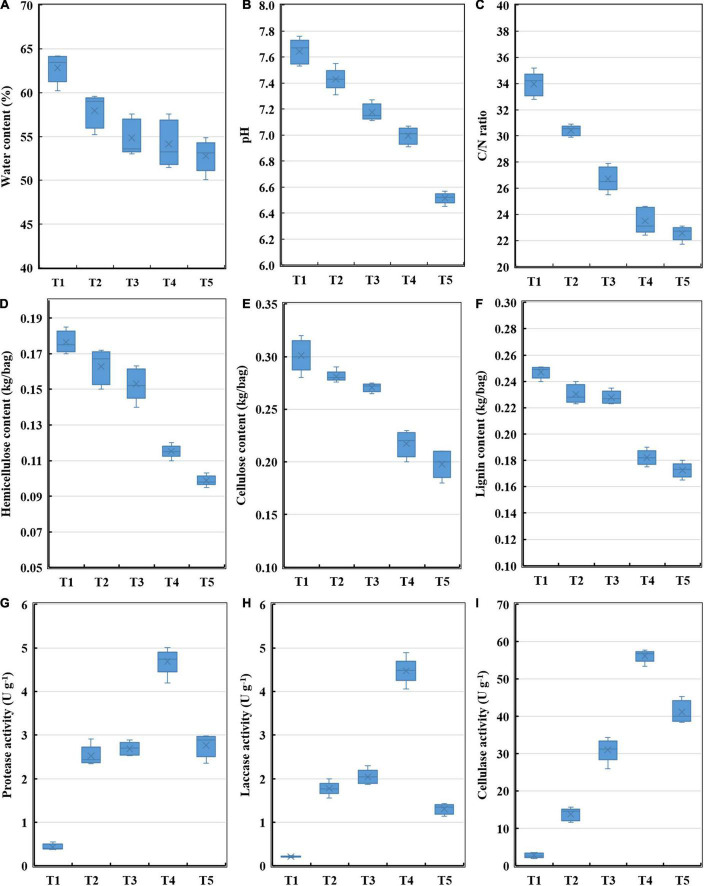
Enzyme activities and physicochemical properties of composting substrate at the different growth stages of oyster mushroom. **(A)** Water content; **(B)** pH; **(C)** C/N ratio; **(D)** hemicellulose content; **(E)** cellulose content; **(F)** lignin content; **(G)** protease activity; **(H)** laccase activity; and **(I)** cellulase activity. T1, T2, T3, T4, and T5 indicate the day on which composting was completed (day 0: before spawning), mycelial colonization (day 3), mid-incubation (day 10), end-incubation (day 20: before primordia formation), and first oyster mushroom fructification (day 30: fruiting body development), respectively.

**FIGURE 2:**
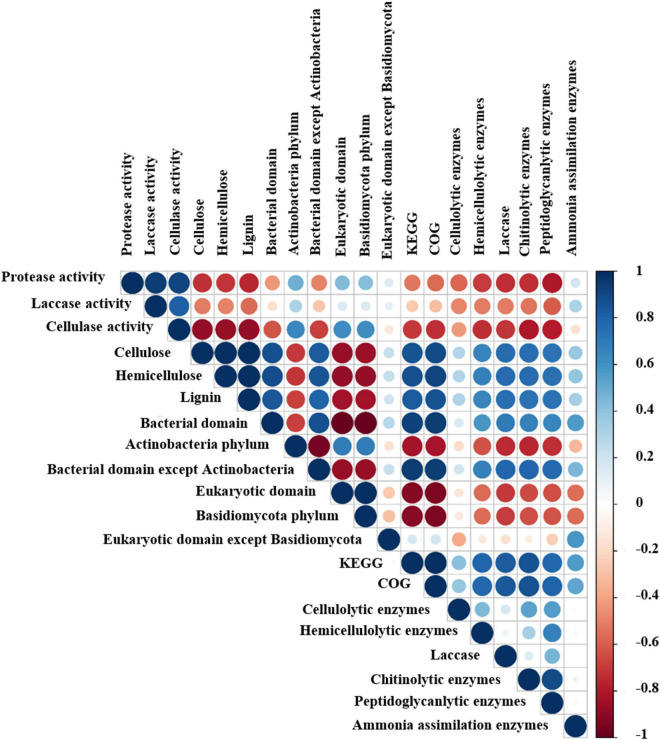
Pearson correlation analysis among the relative abundance of microbial taxa, functional genes and physicochemical properties of the composting substrate at the different growth stages of oyster mushroom. Positive and negative correlations are shown in blue and red, respectively. The size of each circle indicates the value of the correlation coefficient. T1, T2, T3, T4, and T5 indicate the day on which composting was completed (day 0: before spawning), mycelial colonization (day 3), mid-incubation (day 10), end-incubation (day 20: before primordia formation), and first oyster mushroom fructification (day 30: fruiting body development), respectively.

To explore changes in the characteristics of substrate during the oyster mushroom cropping process, the water content, pH, C/N ratio, hemicellulose content, cellulose content, lignin content, protease activity, laccase activity, and cellulase activity were monitored. The water content, pH, C/N ratio, hemicellulose content, cellulose content and lignin content all decrease continuously, while the activities of protease, laccase and cellulase exhibited maximal activity during the end-incubation stage (T4) and decreased thereafter (Figure 1). The hemicellulose content, cellulose content and lignin content rapidly decreased by 42.76, 34.01, and 30.18%, respectively, and were negatively correlated with the activities of protease, laccase and cellulase according to the Pearson correlation analysis (*p* < 0.05) (Figure 2).

### Illumina sequencing of oyster mushroom composting substrate microbiomes

Metagenomics sequencing generated 1.44 × 10^9^ original reads, and each sample was 8.33 × 10^7^–1.25 × 10^8^ reads. The clean reads of a single sample after data quality control accounted for more than 99% of the original reads. A total of 2,652,407 assembly sequences (contigs) were acquired from the metagenomics sequencing; the total sequence length of a single sample contig was 35.74–58.63 Mb, and the N50 was 517–535 bp (Table 1). Prokka v1.13.3 2 was used to predict the ORFs of the spliced contigs, and genes longer than 100 bp were translated into the corresponding amino acid sequences. This experiment yielded a total of 260,000 genes, and their average sequence length was 536.20 bp (340,700–612,700 from each sample).

**TABLE 1:** Summary of *de novo* assembly results of the five time point metagenomic DNA samples from the composting substrate of oyster mushroom.

Sample	Reads			Contig						
	Raw reads	Clean reads	Clean reads rate (%)	Contig number	N50 (bp)	N90 (bp)	Min (bp)	Max (bp)	Average length (bp)	Total length (Mb)
T1	91,362,759	91,107,035	99.7	344,019	527	328	300	219,239	1,045	35.74
T2	86,663,141	86,419,235	99.7	621,412	535	329	300	190,033	929	57.73
T3	92,009,509	91,754,120	99.7	576,877	521	327	300	266,013	939	54.13
T4	107,098,328	106,863,526	99.8	612,086	524	328	300	223,587	960	58.63
T5	102,947,685	102,701,310	99.8	498,013	517	327	300	202,463	933	46.37

T1, T2, T3, T4, and T5 indicate the day on which composting was completed (day 0: before spawning), mycelial colonization (day 3), mid-incubation (day 10), end-incubation (day 20: before primordia formation), and first oyster mushroom fructification (day 30: fruiting body development), respectively. Raw reads, total number of raw sequences; Clean reads, total number of filtered sequences; Clean reads rate (%), the proportion of clean reads in the original sequence (clean reads * 100%/raw reads); Contig number, the number of assembled contigs; N50 (bp), length of contig N50; N90 (bp), length of contig N90; Min (bp), minimum contig length; Max (bp), maximum length of contig; Average length (bp), average length of contig; Total length (bp), The total length of the sample contig.

### Microbial community composition and evolution of oyster mushroom composting substrate

**FIGURE 3:**
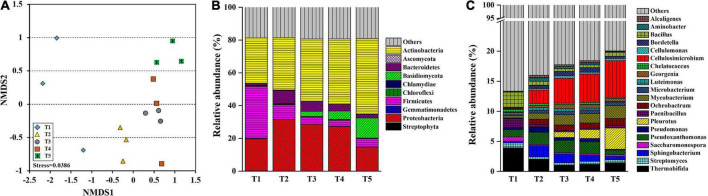
Bray-Curtis-based non-metric multidimensional scaling (NMDS) analysis **(A)** and taxonomic structure of the bacterial community at the phylum **(B)** and genus **(C)** levels in composting substrate at the different growth stages of oyster mushroom. T1, T2, T3, T4, and T5 indicate the day on which composting was completed (day 0: before spawning), mycelial colonization (day 3), mid-incubation (day 10), end-incubation (day 20: before primordia formation), and first oyster mushroom fructification (day 30: fruiting body development), respectively.

Metagenomic sequencing was used to analyze substrate samples at different growth stages of oyster mushroom to examine the genetic information of the microbiota. NMDS ordination was conducted for each growth stage to determine the composition of the microbial community (ANOSIM, *R* = 0.704). The taxa were divided into four distinct groups: T1, T2, and T5 comprised independent groups, while T3 and T4 were clustered into a group (Figure 3A). A total of 44 phyla were identified, of which 35 were in the bacterial domain, 2 were in the archaeal domain, 5 were in the eukaryotic domain, and 2 were of unknown origin. The bacterial domain was predominant (68.96–81.59%), but its abundance gradually decreased by more than 15% overall (Table 2).

**TABLE 2:** Taxonomic origin of predicted genes at domain level in the metagenomes of the five time point composting substrate of oyster mushroom.

Taxon level	Archaea (%)	Bacteria (%)			Eukaryota (%)			Unclassified organisms (%)
		Total	Actinobacteria	Others	Total	Basidiomycota	Others	
T1	0.026	81.592	27.481	54.112	0.023	0.003	0.020	0.002
T2	0.015	80.944	31.761	49.182	0.635	0.238	0.397	0.001
T3	0.013	78.076	39.973	38.103	3.325	3.158	0.167	0.001
T4	0.014	76.166	38.859	37.307	5.225	5.108	0.117	0.001
T5	0.009	68.955	46.214	22.740	12.258	12.179	0.079	0.000

T1, T2, T3, T4, and T5 indicate the day on which composting was completed (day 0: before spawning), mycelial colonization (day 3), mid-incubation (day 10), end-incubation (day 20: before primordia formation), and first oyster mushroom fructification (day 30: fruiting body development), respectively.

Among the top 10 most abundant phyla identified during oyster mushroom cropping, 8 phyla belonged to the bacterial domain, and 2 phyla belonged to the eukaryotic domain. The 8 bacterial phyla were, according to abundance from high to low, Actinobacteria, Proteobacteria, Firmicutes, Bacteroidetes, Chloroflexi, Chlamydiae, Gemmatimonadetes, and Streptophyta. The abundance of the Actinobacteria phylum alone continued to increase (by 68%) during oyster mushroom cropping, whereas the abundance of the Bacteroides and Proteobacteria phyla increased from T1 to T2 and then decreased, and the abundance of the other phyla in the community decreased during mushroom cropping (Figure 3B). The 2 eukaryotic phyla were Basidiomycota and Ascomycota. Basidiomycota was the primary dominant phylum, and its abundance increased 50 times from T2 to T5; the only species in this phylum was oyster mushroom. In contrast, the abundance of Ascomycota increased from T1 to T2 and then decreased (Figure 3B). The abundance of oyster mushroom was significantly positively correlated with that of Actinobacteria (*p* < 0.05), and the total abundance of oyster mushroom and that of Actinobacteria were significantly negatively correlated with the total abundance of the bacterial domains except for Actinobacteria, respectively (*p* < 0.01). Furthermore, the total abundance of oyster mushroom and that of Actinobacteria were significantly negatively correlated with lignin content, hemicellulose content or cellulose content, respectively (*p* < 0.01) (Table 2 and Figure 2).

The top 20 most abundant genera throughout the oyster mushroom cropping period were all bacterial genera except for that of oyster mushroom. There were 8 genera belonging to Actinomycetales, among which the abundance of the 4 genera *Cellulosimicrobium*, *Mycobacterium*, *Saccharomonospora*, and *Microbacterium* gradually increased, whereas that of *Georgenia* and *Cellulomonas* first rose and then decreased, that of *Thermobifida* continuously decreased, and that of *Streptomyces* remained constant. The abundance of the other bacterial genera first increased and then decreased, except for that of *Ochrobactrum*, which continuously increased, and that of *Paenibacillus*, *Chelatococcus*, and *Bacillus*, which continuously decreased (Figure 3C).

### Variation in the composition of the microbial community at different cropping stages

**FIGURE 4:**
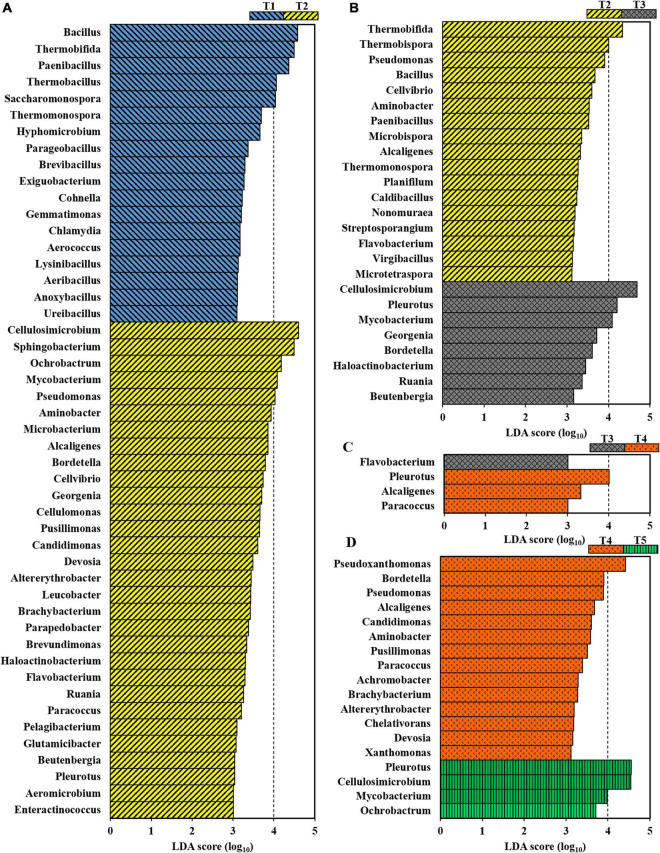
Taxonomic differences at the genus level for bacteria in composting substrate at the different growth stages of oyster mushroom using a linear discriminant analysis (LDA) and effect size (LEfSe) method (LDA threshold = 3.0). **(A)** T1 vs. T2; **(B)** T2 vs. T3; **(C)** T3 vs. T4; and **(D)** T4 vs. T5. T1, T2, T3, T4, and T5 indicate the day on which composting was completed (day 0: before spawning), mycelial colonization (day 3), mid-incubation (day 10), end-incubation (day 20: before primordia formation), and first oyster mushroom fructification (day 30: fruiting body development), respectively.

Bacterial taxa that differed significantly in abundance among the different growth stages of oyster mushroom were identified using a biomarker analysis based on the LDA effect size (LEfSe) method. According to an LDA threshold of 3.0, 48, 25, 4, and 18 bacterial genera showed statistically significant differences between T1 and T2 (Figure 4A), T2 and T3 (Figure 4B), T3 and T4 (Figure 4C), and T4 and T5 (Figure 4D), respectively. This analysis indicated that the community compositions of T1 and T2 differed to the greatest degree, followed by T2 and T3, while T3 and T4 were the most similar. When oyster mushroom grows in the composting substrate, there is a dramatic modification of the native microbiome of the composting substrate, which is most apparent from the significant differences in the composition of the microbial community between T1 and T2. As shown in Figure 4A, 30 genera, including *Cellulosimicrobium*, *Sphingobacterium, Mycobacterium, Pseudomonas* and *Cellvibrio*, were enriched in T2. A comparison of T2 and T3 found 17 genera that were significantly enriched in T2; however, only eight genera were more abundant in T3 (Figure 4B). Genera belonging to *Actinobacteria*, including *Cellulosimicrobium*, *Pleurotus, Mycobacterium* and *Georgenia*, were significantly enriched in T3. Interestingly, thermotolerant bacteria, such as *Thermobifida*, *Thermobispora*, and *Thermomonospora*, were enriched in T2. Additionally, *Pseudomonas* and *Bacillus* were also enriched in T2. Only a limited number of genera differed significantly between T3 and T4 (Figure 4C). Genera such as *Pleurotus, Alcaligenes*, and *Paracoccus* were enriched in T4. Fewer genera were enriched in T5 in comparison with the number of enriched genera in T4 (Figure 4D).

### Microbial metabolism of the composting substrate

**FIGURE 5:**
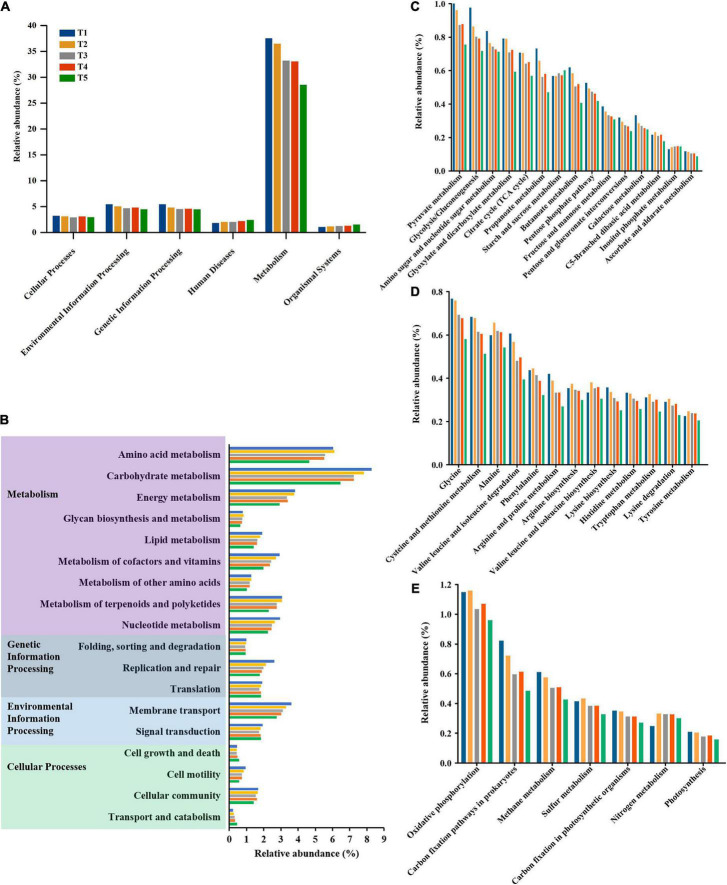
Variation of microbial functional profiles during the different growth stages of oyster mushroom. **(A,B)** Show the level 1 and level 2 KEGG ortholog function annotation, respectively; **(C)** carbohydrate metabolism; **(D)** amino acid metabolism; **(E)** energy metabolism. T1, T2, T3, T4, and T5 indicate the day on which composting was completed (day 0: before spawning), mycelial colonization (day 3), mid-incubation (day 10), end-incubation (day 20: before primordia formation), and first oyster mushroom fructification (day 30: fruiting body development), respectively.

KEGG pathway annotation of the predicted gene sequences obtained from 15 substrate samples enabled the identification of a total of 378 KOs, which were organized into 38 small metabolic pathways and 378 metabolic subsystems. The identified metabolic pathways were associated with six basic metabolic systems (Figure 5A). A total of 3.01–3.33% of the KOs were related to cellular processes, 4.59–5.54% were related to environmental information processing, 4.55–5.54% were related to genetic information processing, 1.94–2.56% were related to human diseases, 28.61–37.63% were related to metabolism, and 1.17–1.64% were related to organismal systems (Figure 5A). The metabolic pathways for carbohydrates, amino acids and energy were the primary pathways activated during the process of oyster mushroom cropping (Figure 5B). The most enriched pathways, ranked from highest to lowest level of enrichment, were pyruvate metabolism, glycolysis/gluconeogenesis and amino sugar and nucleotide sugar metabolism in carbohydrate metabolism (Figure 5C). The top 3 enriched pathways were glycine metabolism, cysteine and methionine metabolism, and alanine metabolism in amino acid metabolism (Figure 5D). The top 3 most enriched pathways in energy metabolism were oxidative phosphorylation, carbon fixation pathways in prokaryotes and methane metabolism (Figure 5E).

### Dynamic succession of the functional genes in clusters of orthologous groups of proteins

**FIGURE 6:**
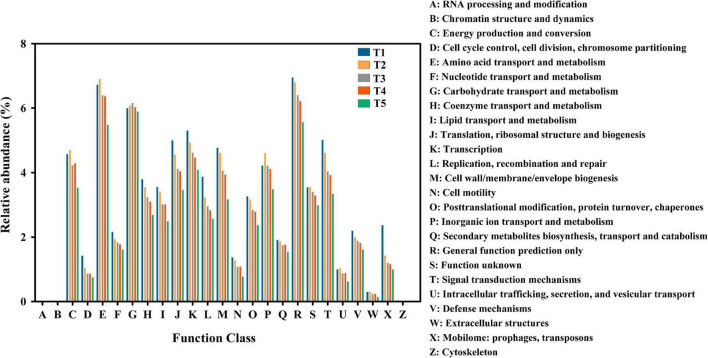
Cluster of Orthologous Groups of proteins (COG) classification. The abscissa is the COG entry, and the ordinate is the relative abundance of COG genes with different functions in composting substrate at the different growth stages of oyster mushroom. T1, T2, T3, T4, and T5 indicate the day on which composting was completed (day 0: before spawning), mycelial colonization (day 3), mid-incubation (day 10), end-incubation (day 20: before primordia formation), and first oyster mushroom fructification (day 30: fruiting body development), respectively.

A total of 4,304 COG genes were identified in 15 samples. The top 5 pathways with high abundance were R (general function prediction only), E (amino acid transport and metabolism), G (carbohydrate transport and metabolism), K (transcription), and C (energy production and conversion) (Figure 6). Pearson correlation analysis indicated that total COG abundance was positively correlated with total KEGG abundance during different stages of oyster mushroom cropping (*p* < 0.0001) (Figure 2).

**FIGURE 7:**
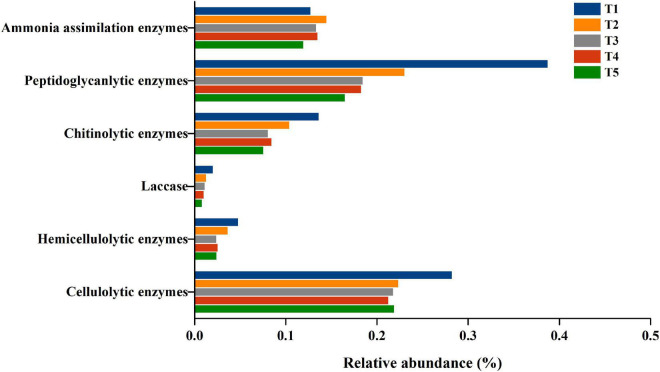
The relative abundance of COG genes with cell wall degradation functions in composting substrate at the different growth stages of oyster mushroom. T1, T2, T3, T4, and T5 indicate the day on which composting was completed (day 0: before spawning), mycelial colonization (day 3), mid-incubation (day 10), end-incubation (day 20: before primordia formation), and first oyster mushroom fructification (day 30: fruiting body development), respectively.

Furthermore, the genes of cellulolytic enzymes, hemicellulolytic enzymes, laccase, chitinolytic enzymes, peptidoglycanlytic enzymes and ammonia assimilation enzymes identified in the eggNOG database were all aligned to the COG database. The cellulolytic enzyme genes included endoglucanase (EC 3.2.1.4) (COG3405), β-glucosidases (EC 3.2.1.21) (COG1472 and COG2723), cellobiose phosphorylase (2.4.1.20) (COG3459), and 6-phospho-β-glucosidase (EC 3.2.1.86) (COG1486). The hemicellulolytic enzyme genes were xylanase (COG3693) and mannanase (COG4124). Laccase genes were all classified in COG1496. The chitinolytic enzyme genes were chitinase (COG3469), N-acetylglucosaminidase (COG4724), chitin deacetylase (COG0726), and lysozyme (COG3772). The peptidoglycanlytic enzyme genes were peptidoglycan hydrolase (COG3883, COG1705, COG3409, COG1619, and COG2385), peptidoglycan/xylan/chitin deacetylase, PgdA/CDA1 family (COG0726), and endo-beta-N-acetylglucosaminidase D (COG4724, COG3757). The genes encoding ammonia assimilation enzymes included glutamate dehydrogenase (EC1.4.1. X) (COG0334), glutamine synthetase (EC6.3.1.2) (COG0174) and glutamate synthase (EC1.4.1.13) (COG006). The abundance of the 6 groups of genes gradually decreased, except for the genes encoding ammonia assimilation enzymes throughout oyster mushroom cropping, and the gene abundance of ammonia assimilation enzymes increased from T1 to T2 and then decreased (Figure 7).

Pearson correlation analysis revealed that the gene abundance of cellulolytic enzymes was positively correlated with that of chitinolytic enzymes and peptidoglycanlytic enzymes. The gene abundance of hemicellulolytic enzymes, laccase, chitinolytic enzymes and peptidoglycanlytic enzymes ranged from 0.28 to 0.24%, 0.05 to 0.02%, 0.02 to 0.01%, 0.14 to 0.08%, 0.39 to 0.16%, and 0.13 to 0.12% during the mushroom cropping was positively correlated with the hemicellulose content, cellulose content and lignin content in the compost, and positively correlated with the abundance of bacterial domains, except for the Actinobacteria phylum. The gene abundance of ammonia assimilation enzymes was positively correlated with the abundance of the bacterial domain (Figure 2). These results indicated that the metabolic activity of the short composting substrate during oyster mushroom cropping was dominated by bacteria.

## Discussion

Oyster mushroom composting substrate prepared by the short composting method contains a wide variety of microorganisms in high abundance, has high water-soluble organic carbon (DOC) content and ammonia content, and shows a temperature 10°C higher than the ambient temperature during the process of mushroom cropping (Kong et al., 2020, 2021). The most rapid degradation of hemicellulose, cellulose and lignin occurred during T3–T4, which was also the period during which the activity levels of laccase and cellulase increased most rapidly during the process of oyster mushroom cropping with short composting substrates. The hemicellulose content, cellulose content and lignin content were negatively correlated with laccase activity, cellulase activity and Actinobacteria phylum abundance. When oyster mushroom grows on a sterilized substrate, the most rapid lignin degradation occurs at the same as that of a mushroom grown on a short composting substrate, while the most rapid cellulose degradation occurs during the fruiting period (Kong et al., 2016). The activity levels of laccase and cellulase increase most rapidly during the fruiting body induction and rapid growth period (Gao et al., 2017). The period of rapidly increasing lignocellulose-degrading enzyme activity and lignocellulose degradation in oyster mushrooms growing on a short composting substrate occurred earlier than the corresponding period for mushrooms grown on sterilized substrates; this difference may be related to the large number and high diversity of the microorganisms in the short composting substrate, as well as the high temperature of the substrate (Kong et al., 2021).

During the period from oyster mushroom spawning to full colonization on the short composting substrate, the hemicellulose content, cellulose content and lignin content of the compost substrate decreased by 34.15, 28.24, and 26.87%, respectively. In addition, hemicellulose content, cellulose content and lignin content were negatively correlated with the abundance of the Actinobacteria phylum. During the short composting period, 35, 14, and 16% of the hemicellulose, cellulose and lignin in the substrate, respectively, were consumed (data not published). In the same growth phase, the lignin content of the button mushroom composting substrate was degraded by 45%, while only a small proportion of the carbohydrates in the substrate was degraded, and xylan was partially degraded (Jurak et al., 2015). However, during phase II of composting, 50% of both xylan and cellulose were metabolized, whereas lignin structures were unaltered (Jurak et al., 2015). These results revealed that the other microbes, especially Actinobacteria, contributed to lignocellulosic degradation on the short composting substrate during oyster mushroom colonization, but not button mushroom colonization (Carrasco et al., 2020).

During oyster mushroom cropping in the short composting substrate, bacteria domain was the absolute dominant component although its abundance was slightly reduced with 15.5%, but the dynamic succession of different groups in the domain was dissimilar. The increase in the abundance of the Actinobacteria phylum is assumed to be related to the continuous increases in the abundance of the 4 genera *Cellulosimicrobium*, *Mycobacterium, Saccharomonospora*, and *Streptomyces*, which showed high abundance and all belonged to the order of Actinomycetales throughout the oyster mushroom cropping period. Members of these 4 genera all show strong cellulose-degrading ability (Berlemont and Martiny, 2013; Větrovský et al., 2014; Talamantes et al., 2016). Another Actinomycetales genus with high abundance and cellulose-degrading ability was *Thermobifida*, whose abundance decreased rapidly, most likely due to the presence of a favorable temperature range for the growth of thermobacteria (35°C–60°C) (Talamantes et al., 2016). Among the other bacterial phyla, the abundance of the facultative anaerobic and strong cellulose-degrading bacteria *Ochrobactrum* increased continuously, while that of the thermophilic bacteria *Paenibacillus* and *Chelatococcus* (Talamantes et al., 2016) and the aerobic bacteria *Bacillus* decreased continuously. The abundance of facultative anaerobic cellulose-degrading bacteria *Sphingobacterium*, *Pseudoxanthomonas*, *Pseudomonas*, *Luteimonas*, *Aminobacter*, *Alcaligenes*, and *Bordetella* increased first and then decreased, and this trend was probably associated with the rapid reduction in the pH of the compost substrate. With the exception of the Basidiomycota phylum, the abundance of eukaryotic domains was low and gradually decreased, primarily because inhibitory substances that formed during compost processing inhibited competitive molds (Cui et al., 2021). Thus, the fate of the bacteria in the short composting substrate used for oyster mushroom cropping was dependent on favorable temperature, oxygen, and pH conditions, as well as their cellulose degradation ability. The relationship between oyster mushrooms and the microorganisms, especially Actinomycetales, in a short composting substrate on which they are grown is expected to be cooperative. The Actinomycetales should be oyster mushroom growth promoting bacteria (OMGPB).

Carbohydrate metabolism, amino acid metabolism and energy metabolism were the primary enriched pathways among the annotated KEGG metabolic pathways of the microbial community of the short composting substrate during the oyster mushroom cropping process. The metabolism of carbohydrates, amino acids and energy accounted for more than 30% of all pathways identified throughout the oyster mushroom cropping process, which may help to explain why the substrate temperature was 10°C higher than the ambient temperature (Kong et al., 2021). In the amino acid metabolism pathway, the top 3 most enriched pathways were glycine metabolism, cysteine and methionine metabolism, and alanine metabolism. Andresen et al. (2014) reported that Gram-positive bacteria preferentially utilize glycine substrate in soil, so the glycine metabolic pathway should be dominated by bacteria. The 3 most abundant pathways related to energy metabolism were oxidative phosphorylation, carbon fixation pathways in prokaryotes, and methane metabolism. Carbon fixation pathways in prokaryotes and methane metabolism only exist in prokaryotes (Evans et al., 2019). Thus, the two pathways were also dominated by bacteria. In addition, the total abundance of COGs from the database for prokaryotic genome annotation and comparative genomics (Galperin et al., 2018) in the short composting substrate during oyster mushroom cropping was positively correlated with the total abundance of KEGG orthologs from the database for cellular and organism-level functions inferred from genome sequences and other molecular virus, prokaryotic and eukaryotic datasets (Kanehisa et al., 2021). The genes encoding cellulolytic enzymes, hemicellulolytic enzymes, laccase, chitinolytic enzymes, peptidoglycanlytic enzymes and ammonia assimilation enzymes identified in the eggNOG database, used for nested orthology inference across eukaryotic and prokaryotic genomes (Powell et al., 2014), were all aligned to the COG database. The gene abundance of hemicellulolytic enzymes, laccase, chitinolytic enzymes, and peptidoglycanlytic enzymes was positively correlated with the abundance of bacterial domains except for the Actinobacteria phylum. The gene abundance of ammonia assimilation enzymes was positively correlated with the abundance of bacterial domains. These results indicated that the metabolic activity of the short composting substrate during oyster mushroom cropping was dominated by bacteria.

The button mushroom is a typical secondary decomposer of lignocellulose, and oyster mushroom is a typical primary decomposer of lignocellulose (McGee et al., 2017). During button mushroom colonization of compost, the abundance of bacteria in compost decreased except for *Pseudomonas*, and *Scytalidium*, the most abundant fungal genus, almost disappeared (Carrasco and Preston, 2020; Carrasco et al., 2020). Bacterial biomass nearly three-fold reduction, other fungi were dead (Vos et al., 2017; Carrasco et al., 2020). The reduction of these bacteria and fungi is due to their degradation by button mushroom (Fermor and Wood, 1991; Fermor et al., 1991). Therefore, the biomass of button mushroom largely derives from the degradation of other bacteria and fungi in compost. However, during oyster mushroom colonization of compost, the abundance of bacteria decreased less, and the abundance of Actinobacteria also increased remarkably. Thus, many nutrients were also stored in bacterial cells, which was not conducive to the improvement of the yield and quality of oyster mushrooms. This may be an important difference between primary and secondary degraders of lignocellulose. Increasing the degradation and utilization of oyster mushroom to bacteria should raise the mushroom yield and quality.

## Conclusion

As far as we know, this study firstly revealed the dynamic succession of microbial compost communities and functions, and the relationship between the microorganisms resided in compost and oyster mushroom during the mushroom cropping on a short composting substrate. The dominant flora in the compost was bacteria, followed by fungi. The abundance of Actinobacteria phylum increased while decreased in other bacteria. Fungi was almost comprised of oyster mushroom. The abundance of Actinobacteria was positively correlated with that of oyster mushroom, positively correlated with the lignocellulose-degrading enzyme activities in the compost, and negatively correlated with the lignocellulose contents. The predicted major functional genes, metabolic pathways and enzyme genes were also derived from bacteria. These results indicated that bacteria, especially Actinomycetales, were the main metabolism participants in the compost during the oyster mushroom cropping. The relationship between oyster mushrooms and bacteria was cooperative, Actinomycetales were OMGPB. Enhancing the decomposition ability of oyster mushrooms over that of microorganisms resided in compost will likely increase the mushroom yield.

## Data availability statement

All the raw metagenomic datasets are available at the National Genomics Data Center (NGDC) Genome Sequence Archive under the accession number: CRA003534 and is publicly accessible at https://bigd.big.ac.cn/gsa.

## Author contributions

QL, WK, YZ, and LQ designed the study. QL, XC, ZS, JW, and SH prepared the composting substrate and processed samples for metagenomic sequencing. QL, XC, and WK performed the bioinformatic analyses. QL and WK drafted the manuscript. QL and LQ revised the manuscript. All authors reviewed and approved the final manuscript.
